# Women’s knowledge and attitudes to the menopause: a comparison of women over 40 who were in the perimenopause, post menopause and those not in the peri or post menopause

**DOI:** 10.1186/s12905-023-02424-x

**Published:** 2023-08-30

**Authors:** Bisma Tariq, Samantha Phillips, Rina Biswakarma, Vikram Talaulikar, Joyce C Harper

**Affiliations:** 1 Institute for Women’s Health, University College London, London, UK; 2 Institute for Education, University College London, London, UK; 3 Reproductive Medicine Unit, University College Hospital, London, UK

## Abstract

**Objectives:**

To evaluate women’s knowledge and attitudes towards the menopause by comparing three groups of women: perimenopause, post menopause and those women not in either the peri or post menopause (other).

**Methods:**

A 35 question online survey was advertised on social media to evaluate women’s attitudes and knowledge of the menopause. Three groups of women were compared: perimenopause, post menopause and those women not in either the peri or post menopause (other).

**Results:**

Most women were completely uninformed or only had some knowledge of the menopause before the age of 40. Most women thought that the menopause should be taught at school, but over 80% had received no menopause education at school themselves. The most popular sources of menopause information were independent websites and friends. Perimenopausal women were significantly more likely than postmenopausal women to use online resources for menopause information. The perimenopausal and postmenopausal groups had more positive attitudes towards the menopause than the other group. 57.5% of postmenopausal women found the menopause difficult or very difficult. Most women were happy about no longer menstruating, although some expressed sadness regarding fertility loss.

**Conclusions:**

Most women had limited knowledge and negative attitudes towards the menopause, leaving them unprepared to cope with the physical and psychological changes associated with this stage of life. Improved menopause education is required to improve quality of life during the menopausal transition and a most positive narrative of life postmenopause.

**Supplementary Information:**

The online version contains supplementary material available at 10.1186/s12905-023-02424-x.

## Introduction

Despite the fact that almost every woman will go through the menopause, there is a negative narrative of the menopause, considerable lack of knowledge within the general population and a lack of training in medical schools [1]. This means that many women are anxious of the menopause, associating it with negativity and doctors are unable to recognise menopause symptoms, which can lead to major effects on wellbeing. This is a major concern, as women who experience menopausal symptoms have a significantly lower, health-related quality of life [2].

We have conducted two surveys to ask women their knowledge and attitudes to the menopause. In our survey of women under 40 years old, we found that many women under 40 had limited education on the menopause [3]. In our survey of women over 40, we have reported the views of perimenopausal and postmenopausal women [4, 5]. In both studies we found that women reported a lack of education and their healthcare professionals’ lack of adequate training on the menopause. This resulted in many women entering this critical life stage with a lack of knowledge, support and appropriate healthcare.

Unsurprisingly, a significant proportion of women desire more information about the menopause [4, 6], and health education interventions have been shown to improve women’s attitudes towards the menopause [7]. For this reason, it is important to ensure that women have a sufficient understanding of the menopause, as it is a process that almost every woman will experience. The European Menopause Survey, showed that women obtained information mainly from non-medical sources such as magazines and television which may not always be correct [8]. The Asian Menopause Survey showed that 21% of women obtained information from friends, 13% from physicians, and lastly from print and electronic media [9].

Attitudes to the menopause are generally negative, most likely worsened due to lack of education and misinformation [10]. Hickey and colleagues “argue that social and cultural attitudes contribute to the varied experience of menopause and that medicalisation fuels negative perceptions”. They discuss that the menopause is a natural event, which we should normalise, emphasising the positive aspects such as freedom from menstruation, pregnancy, and contraception. The narrative that the menopause is a disease that can only be treated by hormone replacement therapy (HRT) may not be helpful to women.

It has been shown that women with more negative attitudes towards the menopause in general report more symptoms during the perimenopause [11]. Menopause-related symptoms have been found to be less prevalent in countries where menopause is viewed as a normal aging process rather than a disease. Globally societies have different attitudes towards menopause which influences how women experience it [12]. The European Menopause Survey revealed that women in the UK had more severe symptoms and poorer quality of life compared to postmenopausal women in Spain and France [8]. In contrast, the Asian Menopause Study revealed that women may leave menopause-related symptoms untreated due to the belief that menopause is a natural life process and are likely to choose alternative methods for symptom relief such as natural remedies or herbal medicines [9].

The aim of this project was to explore and compare women’s knowledge and attitude towards the menopause using an online survey. The detailed analysis of the perimenopause [4] and postmenopause [5] have already been reported. In this paper, we report the comparison of the data between perimenopausal, postmenopausal women and those who were neither peri or post menopause (other) to determine similarities and differences in attitude and knowledge. It is hoped this data will help develop menopause education.

## Methods

All methods were carried out in accordance with relevant guidelines and regulations or declaration of Helsinki. Informed consent was obtained from all study participants.

All experimental protocols were approved by UCL Research Ethics Committee ID no: 9831/005. The sample population for the survey was English-speaking women aged 40 or above at the time of participating in the survey.

Harper et al. (2022) designed an online survey to evaluate women’s knowledge and attitudes towards the menopause [4, 5]. It was generated using the computer software Qualtrics XM® and consisted of 35 questions (Additional File 1). The inclusion criterion was any woman aged 40 or above. In the first section, the following sociodemographic information was collected: age, gender identity, sexual orientation, current relationship status, number of children, highest educational qualification, field of work/study/trade, religion, ethnic identity, and disability status. The second section evaluated the women’s experiences, attitudes and knowledge of the perimenopause and menopause.

In this study, participants were divided into three groups (perimenopause, post menopause and other) based on their response to the question ‘Which of the following stages do you think best describes you?’ Other included women who selected ‘I am not sure if I am perimenopausal/menopausal,’ ‘I do not currently have periods so I am not sure whether I am perimenopausal/menopausal’ and ‘Not in the perimenopause/menopause.’

Using the software IBM SPSS Statistics 27, responses from the three groups were compared and statistical significance was determined using a chi-squared test with a p value of 0.05.

The following survey questions were compared for the three groups:

### Menopause knowledge and education

- Before the age of 40, how informed did you feel about perimenopause/menopause? (Q22)
- What age were you when you started to think about the perimenopause/menopause? (Q23)
- When do you think the menopause should be taught? (Q20)
- How were you taught about the menopause at school? (Q21)
- Have you specifically looked for information of the menopause in any of these ways? (Q24)

### Management of symptoms

- Have you spoken with a health professional about the perimenopause/menopause? (Q27)
- Have you used any of these methods to alleviate perimenopausal/menopausal symptoms? (Q28)

### Attitudes towards the menopause

- How do you feel about the perimenopause/menopause? (only asked to the perimenopause and other groups)(Q14).
- Before you went through the menopause, how did you feel about it? (only asked to the post menopause group)(Q16).
- Now that you have been through the menopause, how do you feel about it? (only asked to the post menopause group)(Q17).
- What are your thoughts about no longer having periods? (Q15)

The answers for Q14 and Q16 were combined for statistical analysis.

Respondents who answered ‘Other’ for Q15 were given the opportunity to enter a free text answer elaborating on their thoughts, and qualitative thematic analysis was carried out on these responses to identify themes.

Since Q17 was only asked to the post menopause group, no statistical analysis was carried out.

### Data Availability

The dataset generated during the current study are not publicly available due to limitations of UCL ethics committee.

## Results

### Sociodemographic characteristics

A total of 3149 women started the survey and 6 were removed as the respondents were below 40. This left 3143 responses for analysis, and incomplete responses were included. The demographics of the women who were included in the study are summarised in Table 1. 950 described themselves as perimenopausal, 934 as postmenopausal and 1049 were classed as other. The average age of all the women in the study was 50.5 years. The majority of respondents identified as female (2867/2940, 97.5%), heterosexual (2666/2940, 90.7%) and white (2562/2705, 94.7%).

Significant differences were found in age, relationship status and highest educational qualification between the three groups (p < .05). Unsurprisingly, due to the timing of menopause later in life, the post menopausal group had the highest mean age (56.5 years), followed by the perimenopausal group (48.1 years) and then the other group (47.4). Being significantly older, the post menopausal group were also more likely to be widowed. No significant differences were found in country of residence, gender identity, sexual orientation, parity or ethnicity.

**Table 1:** Demographic characteristics of the three groups of women in the study

	Perimenopause		Post menopause		Other	
**Age**	**n**	**Frequency (%)**	**n**	**Frequency (%)**	**n**	**Frequency (%)**
40–45	232	24.4	17	1.8	430	41.0
46–51	552	58.1	139	14.9	381	36.3
52–55	149	15.7	275	29.4	188	17.9
> 55	17	1.8	503	53.9	48	4.6
**Country of residence**						
UK	833	87.7	807	86.4	910	86.7
Other	117	12.3	127	13.6	139	13.3
**Gender identity**						
Female	922	97.1	912	97.6	1027	97.9
Non-binary	2	0.2	1	0.1	5	0.5
Other	9	0.9	11	1.2	6	0.6
Prefer not to say	17	1.8	10	1.1	11	1.0
**Sexual orientation**						
Heterosexual	854	89.9	858	21.9	949	90.5
Homosexual	23	2.4	32	3.4	23	2.2
Bisexual	42	4.4	21	2.2	41	3.9
Pansexual	10	1.1	2	0.2	12	1.1
Asexual	3	0.3	4	0.4	4	0.4
Prefer not to say	18	1.9	17	1.8	20	1.9
**Relationship status**						
Single	126	13.3	149	16.0	147	14.0
In a relationship not cohabiting	39	4.1	29	3.1	50	4.8
In a relationship cohabiting	129	13.6	106	11.3	139	13.3
Married/civil partnership	634	66.7	597	63.9	678	64.6
Widowed	13	1.4	28	3.0	17	1.6
Other	5	0.5	13	1.4	11	1.0
Prefer not to say	4	0.4	12	1.3	7	0.7
**Parity**						
1	188	19.8	178	19.1	167	15.9
2	394	41.5	351	37.6	429	40.9
3	117	12.3	117	12.5	138	13.2
4 or more	33	3.5	45	4.8	38	3.6
I do not have children	216	22.7	239	25.6	274	26.1
Prefer not to say	2	0.2	4	0.4	3	0.3
**Highest educational qualification**						
Secondary school	41	4.3	64	6.9	41	3.9
A Level/College level	110	11.6	137	14.7	129	12.3
University undergraduate	222	23.4	169	18.1	236	22.5
University postgraduate	500	52.6	431	46.1	512	48.8
Other	20	2.1	44	4.7	35	3.3
Prefer not to say	3	0.3	5	0.5	2	0.2
**Ethnicity**						
White - English / Welsh / Scottish / Northern Irish / British	723	80.7	704	82.8	763	72.7
White - Irish	39	4.4	30	3.5	33	3.1
Any other white background	83	9.3	85	10.0	100	9.5
Black/Black British - African	0	0	5	0.6	3	0.3
Black/Black British - Caribbean	3	0.3	3	0.4	4	0.4
Any other Black/African/Caribbean background	1	0.1	1	0.1	0	0
Asian/Asian British - Indian	13	1.5	4	0.5	12	1.1
Asian/Asian British - Pakistani	0	0	0	0	3	0.3
Any other Asian background	4	0.4	3	0.4	4	0.4
Arab	1	0.1	0	0	0	0
Latino	3	0.3	1	0.1	3	0.3
Mixed ethnic background	19	2.1	8	0.9	19	1.8
Any other ethnic group	2	0.2	3	0.4	4	0.4
Prefer not to say	5	0.6	3	0.4	8	0.8

### Menopause knowledge and education

**Fig. 1:**
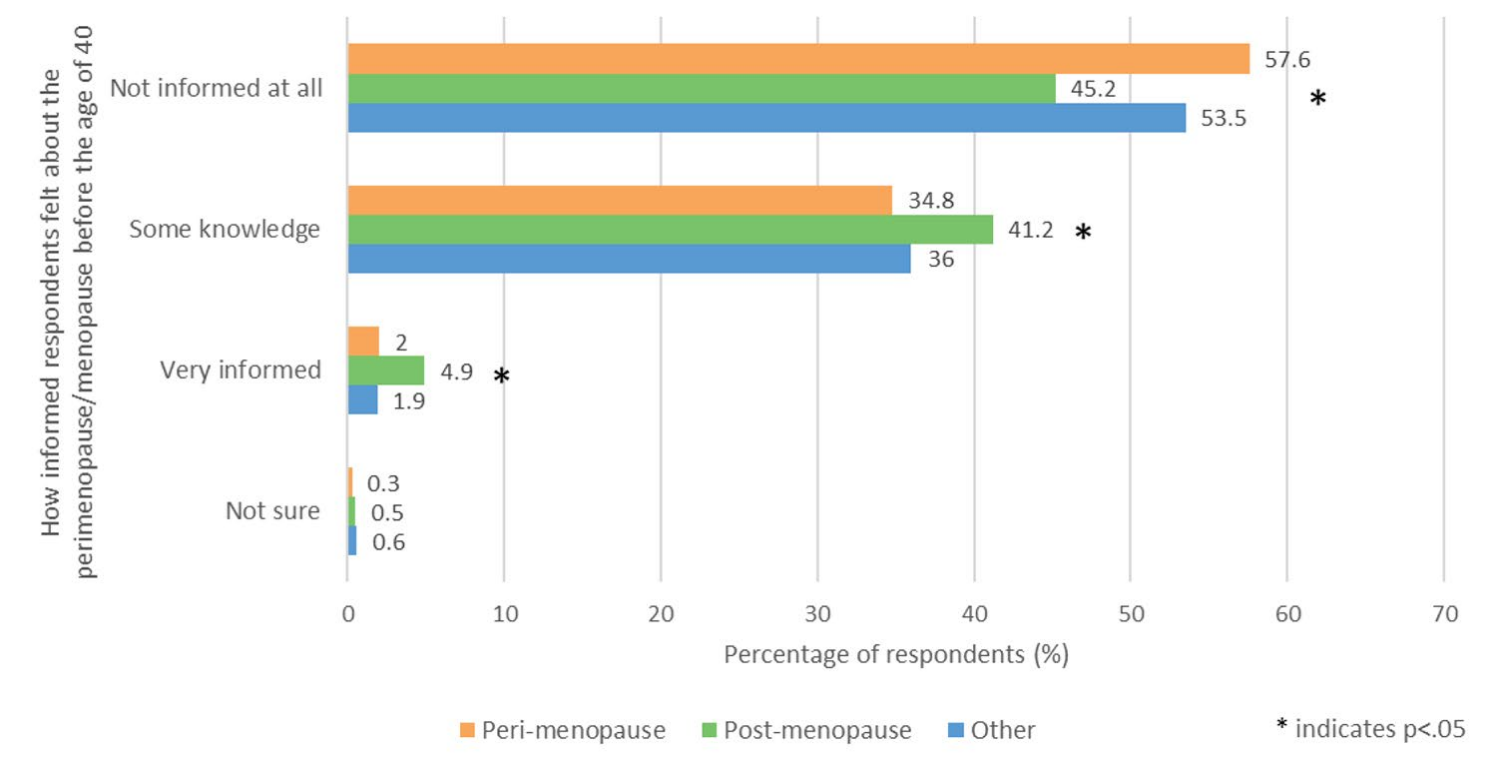
- How informed respondents felt about the perimenopause/menopause before the age of 40

Respondents were asked ‘Before the age of 40, how informed did you feel about perimenopause/menopause?’ (Fig. 1). The majority of the perimenopausal and other groups answered ‘Not informed at all,’ which was significantly greater than the post menopause group; 45.2% (422/934) (p < .05). The post menopause group was significantly more likely to answer ‘Some knowledge’ (41.2%, 385/934) or ‘Very informed’ (4.9%, 46/934) than the other two groups (p < .05).

**Fig. 2:**
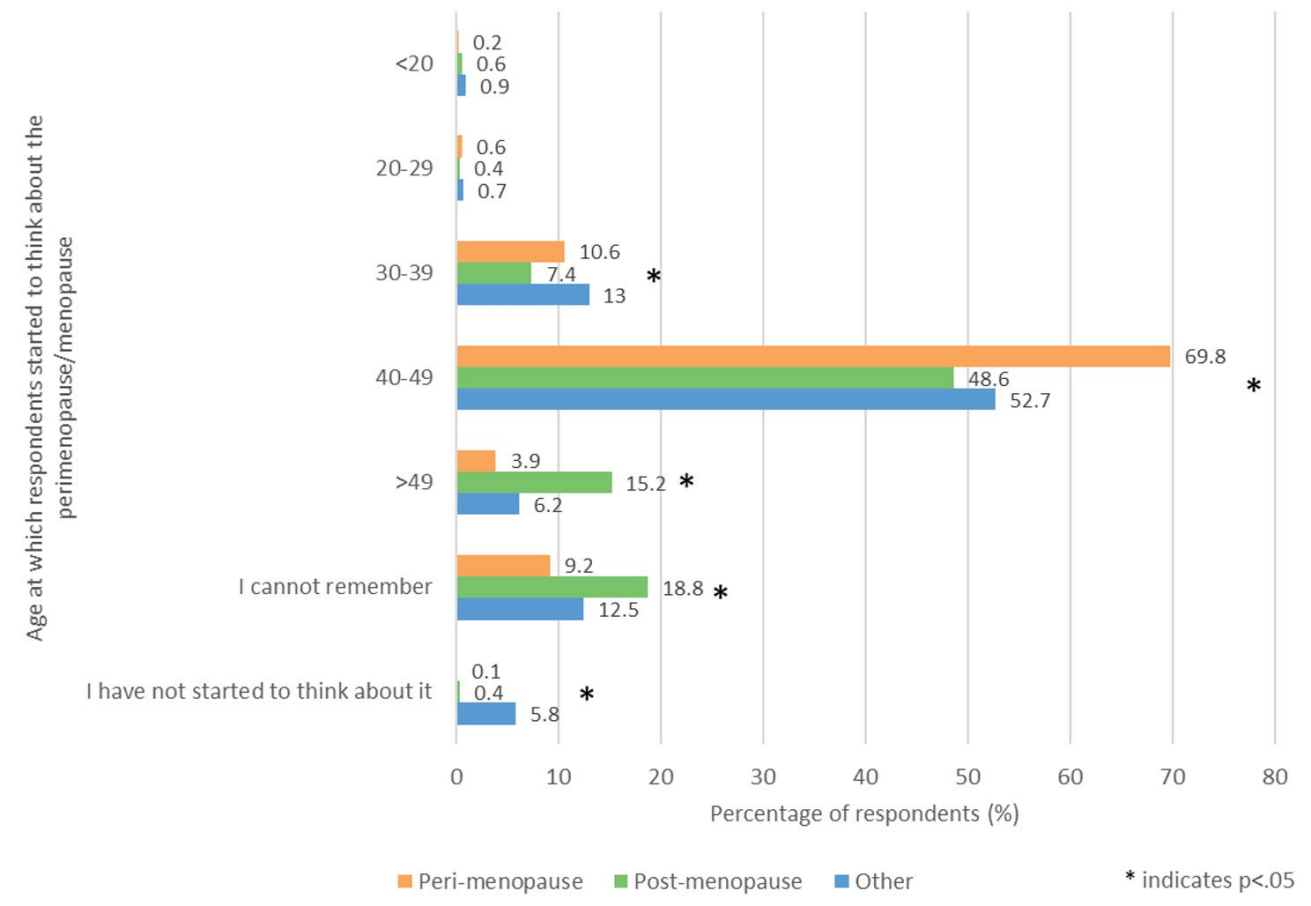
- Age at which respondents had started to think about the perimenopause/menopause

Respondents were asked ‘What age were you when you started to think about the perimenopause/menopause?’ and grouped into five age categories (Fig. 2). The most common age range across all the groups was 40–49, with those in the perimenopause group being significantly more likely than the other two groups to fall into this category (p < .05). Compared with the other two groups, those in the post menopause group were significantly less likely to fall into the 30–39 category, and significantly more likely to fall into the 40–49 or > 49 categories (p < .05). 5.8% (61/1049) of those in the other group answered ‘I have not started to think about it,’ significantly more than in the other two groups (p < .05).

**Fig. 3:**
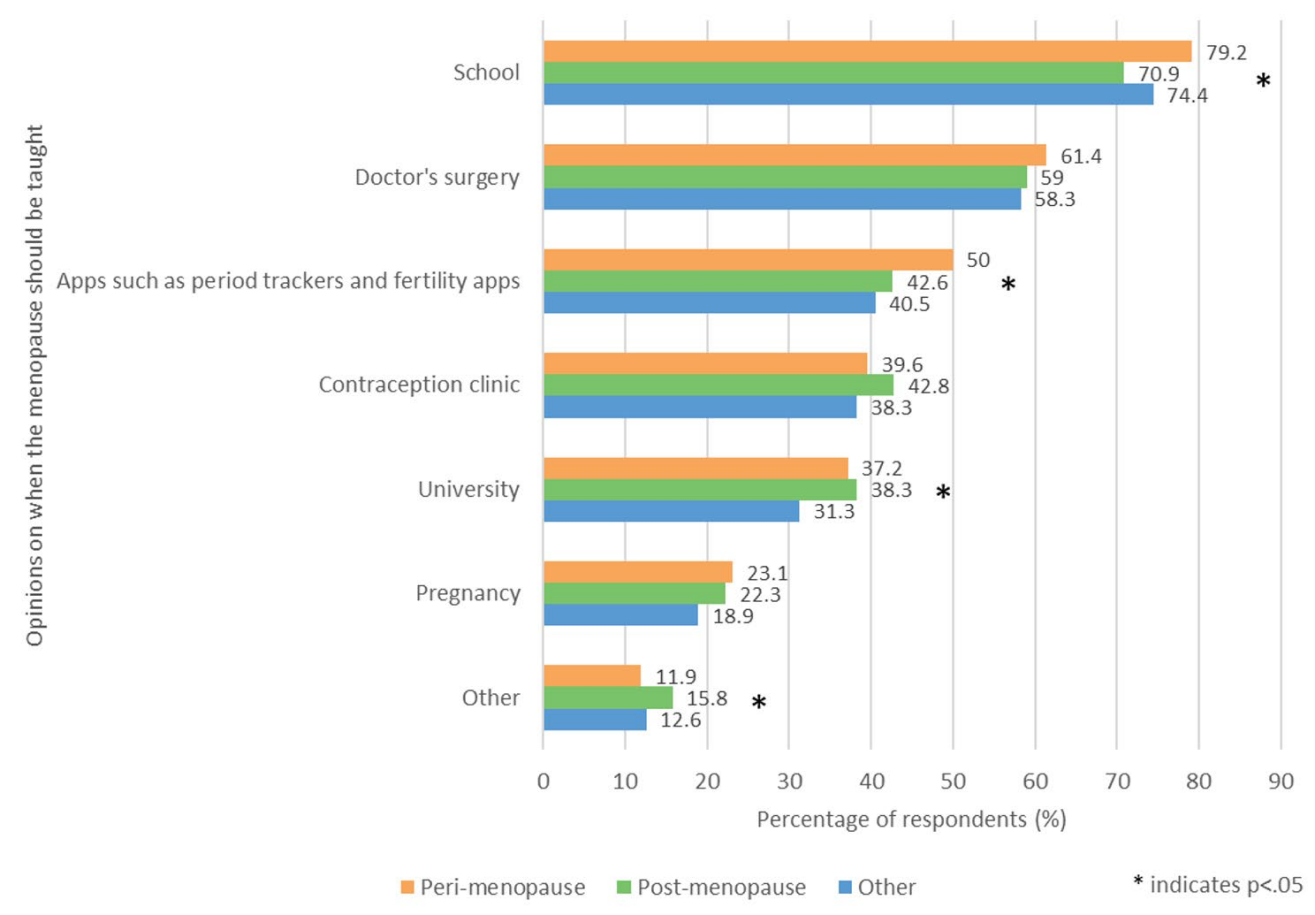
- When respondents thought the menopause should be taught

Respondents were asked ‘When do you think the menopause should be taught?’ and could select multiple answers from the list provided (Fig. 3). The most commonly selected answer for all three groups was ‘School,’ followed by ‘Doctor’s surgery.’ The perimenopause group was significantly more likely to select ‘School’ (79.2%, 752/950) and ‘Apps such as period trackers and fertility apps’ (50%, 475/950) than the other two groups (p < .05). The other group was significantly less likely to select ‘University’ (31.3%, 328/1049) than the other two groups (p < .05). The mean number of total answers selected by each participant was 3.0 in the perimenopausal group, 2.9 in the postmenopausal group and 2.7 in the other group; there was a significant difference between the mean number of total answers selected in the perimenopausal group and the other group (p < .05).

**Fig. 4:**
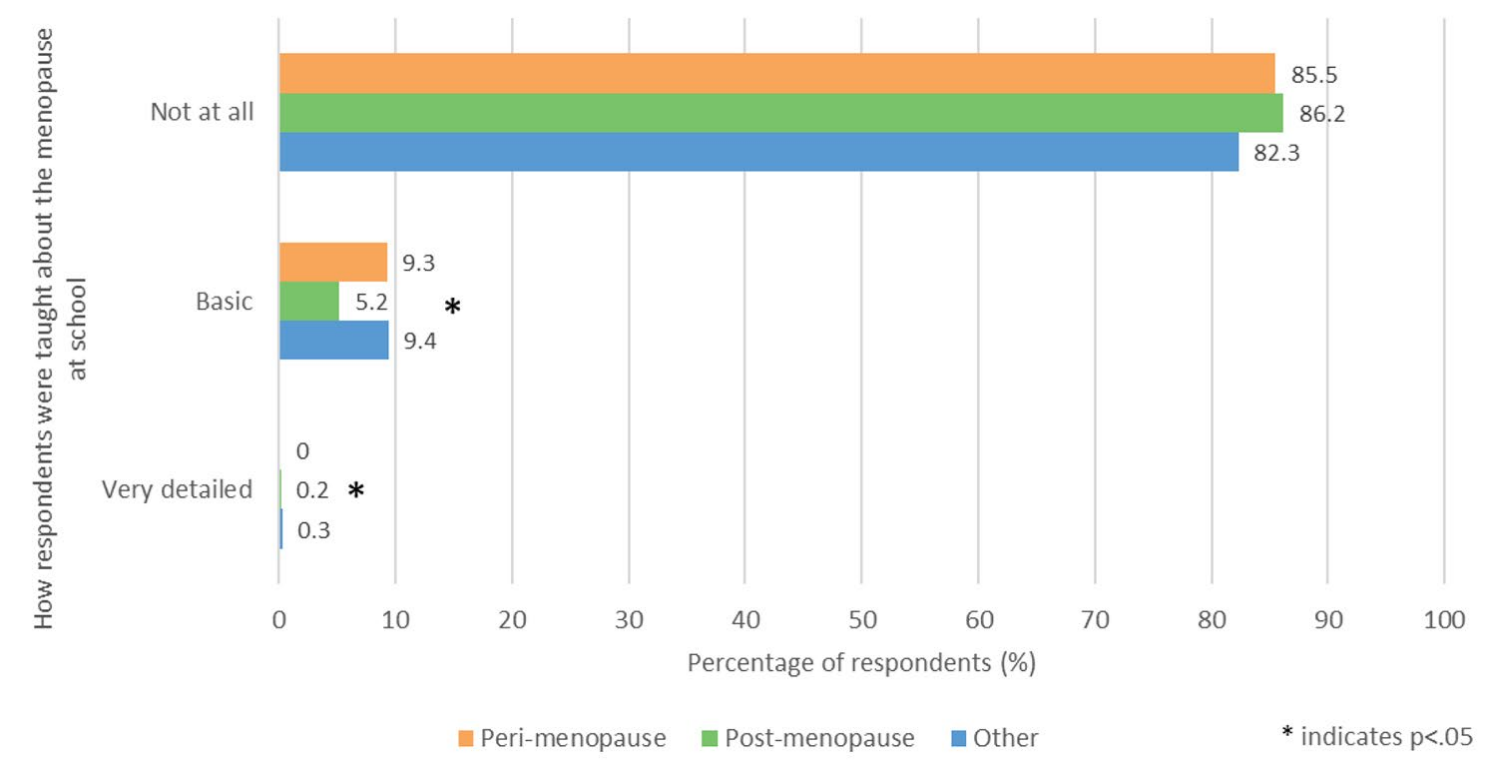
- How respondents were taught about the menopause at school

Respondents were asked ‘How were you taught about the menopause at school?’ (Fig. 4). ‘Not at all’ was the most common answer, being selected by over 80% of participants. The post menopausal group was significantly less likely to answer ‘Basic’ than the other two groups (p < .05), with only 5.2% (49/934) choosing this option compared with 9.3% (88/950) and 9.4% (99/1049) in the perimenopausal and other groups respectively.

**Fig. 5:**
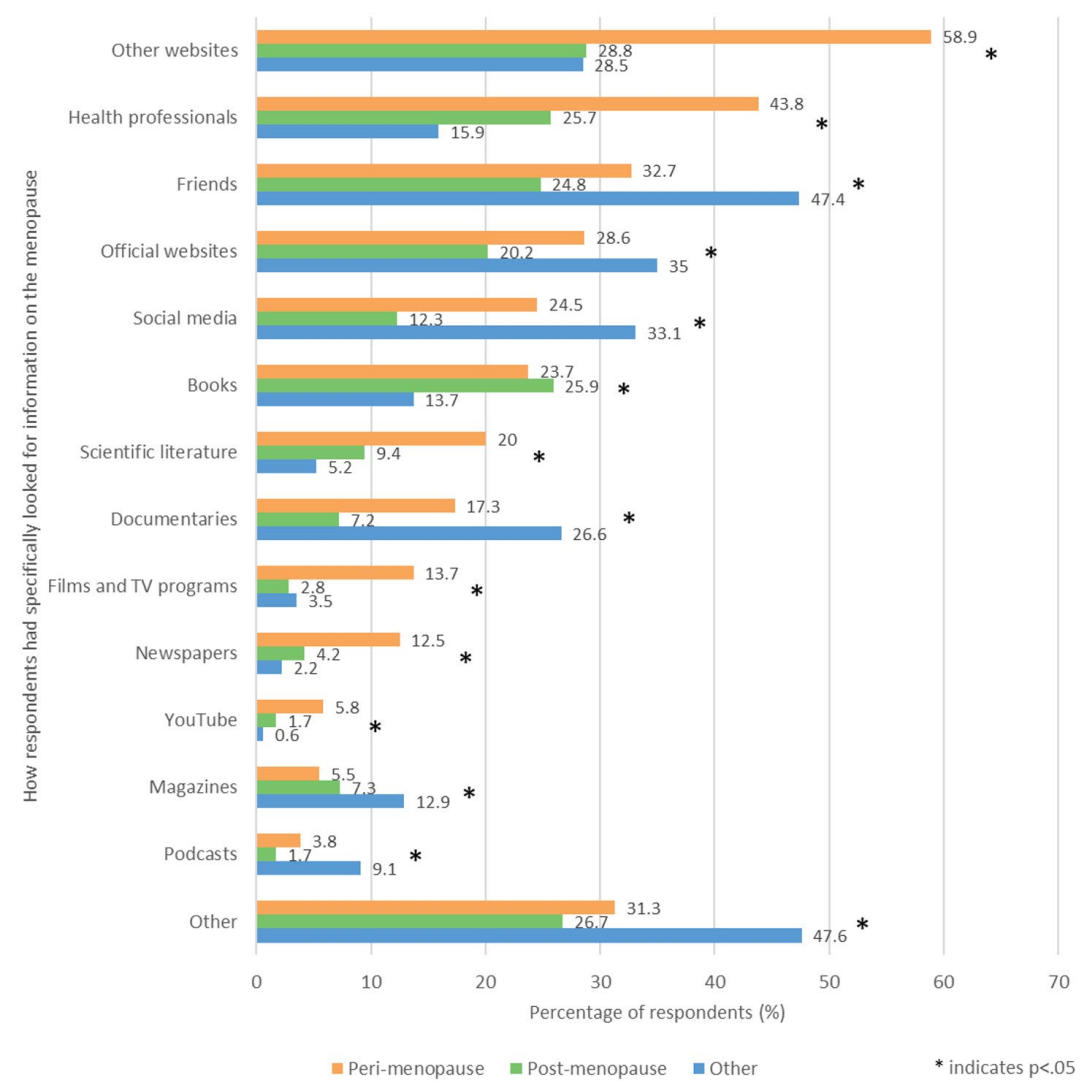
- Ways in which respondents had specifically looked for menopause information

Respondents were asked ‘Have you specifically looked for information of the menopause in any of these ways?’ and could select multiple answers from the list provided (Fig. 5). ‘Other websites’ was the most common answer in the perimenopausal and postmenopausal groups, being selected by 58.9% (560/950) and 28.8% (269/934) respectively. ‘Friends’ was the most common answer in the other group, being selected by 47.4% (497/1049) of respondents. Significant differences were observed between the three groups for all of the answer options, as well as in the mean total number of answers selected (p < .05). The mean total number of answers selected by each participant was 5.2 for the perimenopausal group, 4.3 for the post menopausal group and 3.7 for the other group.

### Management of menopausal symptoms

**Fig. 6:**
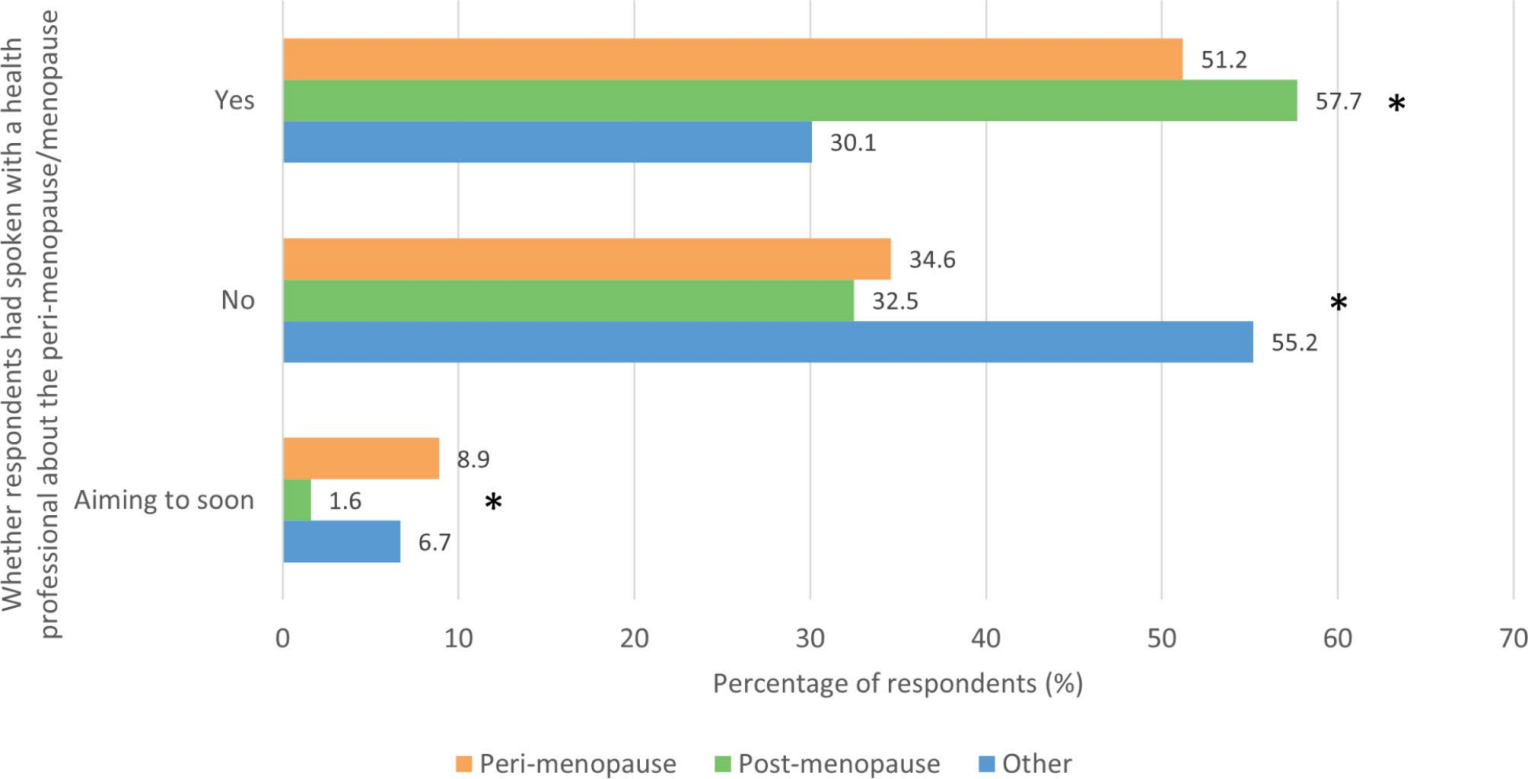
– Respondents were asked if they had spoken with a health professional about the menopause

Respondents were asked ‘Have you spoken with a health professional about the perimenopause/menopause?’ (Fig. 6). 51.2% (486/950) of those in the perimenopausal group and 57.7% (539/934) of those in the post menopausal group answered ‘Yes,’ significantly more than the 30.1% (316/1049) in the other group who chose this answer (p < .05). Conversely, the majority of those in the other group answered ‘No,’ significantly more than the 34.6% (329/950) and 32.5% (304/934) in the perimenopausal and post menopausal groups respectively who chose this answer (p < .05). Those in the post menopausal group were also significantly less likely than those in the other two groups to answer ‘Aiming to soon’ (p < .05).

**Fig. 7:**
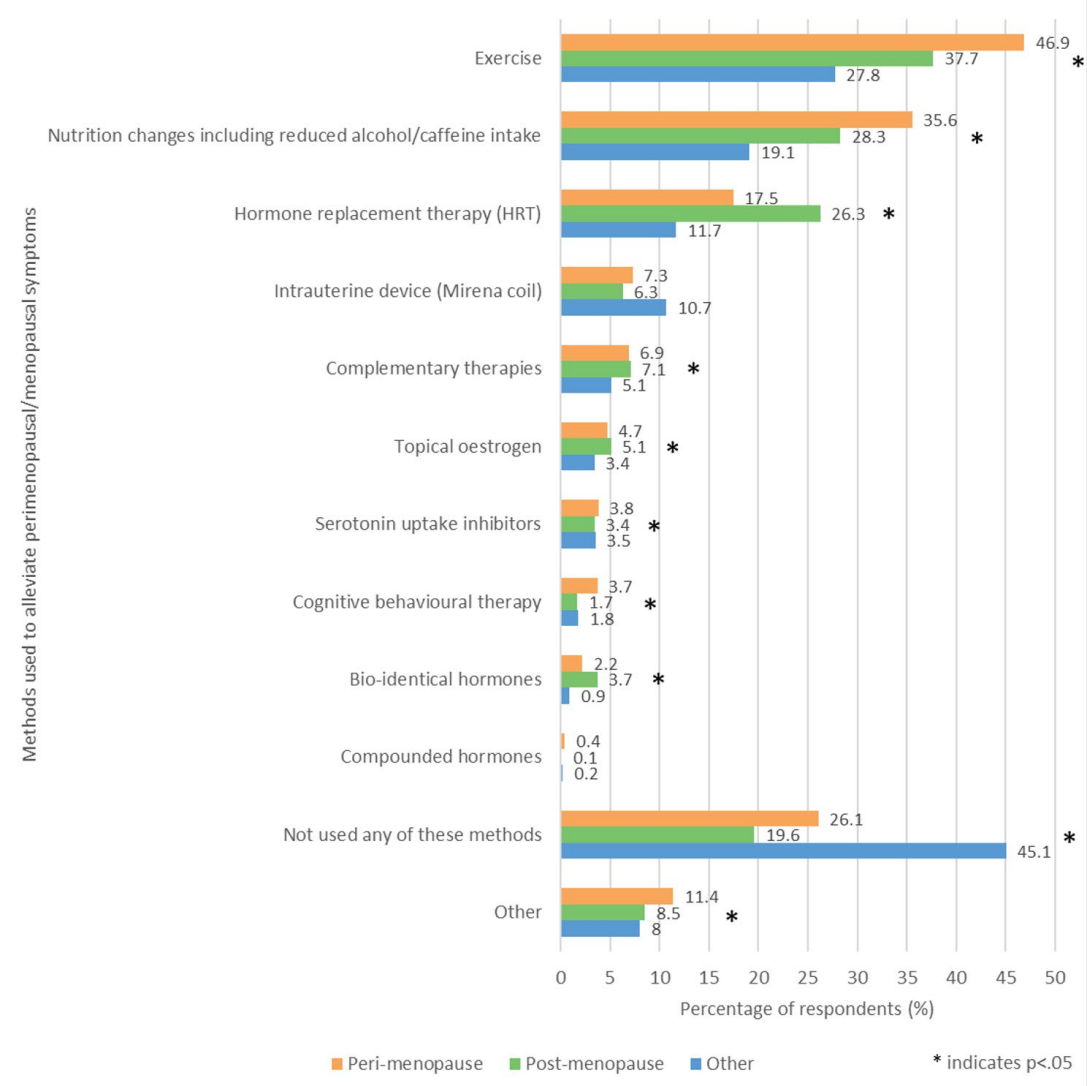
- Methods used to alleviate perimenopausal/menopausal symptoms

Respondents were asked ‘Have you used any of these methods to alleviate perimenopausal/menopausal symptoms?’ and could select multiple answers from the list provided (Fig. 7). ‘Exercise’ was the most common answer in the perimenopausal (46.9%, 446/950) and post menopausal (37.7%, 352/934) groups. ‘Not used any of these methods’ was the most common answer in the other group (45.1%, 473/1049), followed by exercise (27.8%, 292/1049). Other than ‘Compounded hormones’ and ‘Intrauterine device,’ significant differences were observed between the three groups for all of the answer options, as well as in the mean total number of answers selected (p < .05). The mean total number of answers selected by each participant was 2.3 for both the perimenopausal and post menopausal groups, and 2.0 for the other group.

### Attitudes towards the menopause

**Fig. 8:**
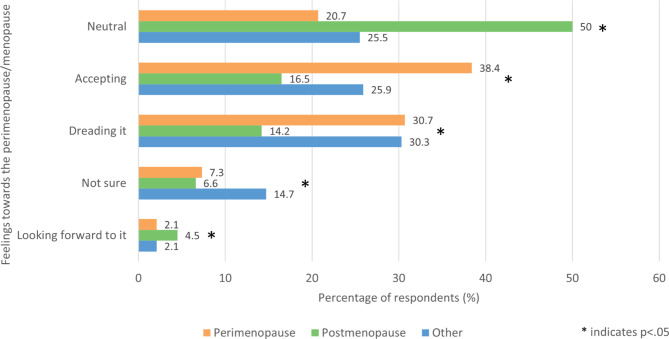
- Feelings towards the perimenopause/menopause

Respondents in the perimenopausal and other groups were asked ‘How do you feel about the perimenopause/menopause?’ and those in the post menopause group were asked ‘Before you went through the menopause, how did you feel about it?’ (Fig. 8). The most common answer was ‘Accepting’ in the perimenopausal group (38.4%, 365/950), ‘Neutral’ in the post menopause group (50%, 467/934) and ‘Dreading it’ in the other group (30.3%, 318/1049). Participants in the post menopausal group were over twice as likely to respond ‘Looking forward to it,’ with 4.5% (42/934) choosing this option compared to 2.1% in the perimenopausal (20/950) and other groups (22/1049). Participants in the post menopausal group were half as likely to respond ‘Dreading it,’ with 14.2% (133/934) choosing this option compared to 30.7% (292/950) and 30.3% (318/1049) in the perimenopause and other groups respectively.

**Fig. 9:**
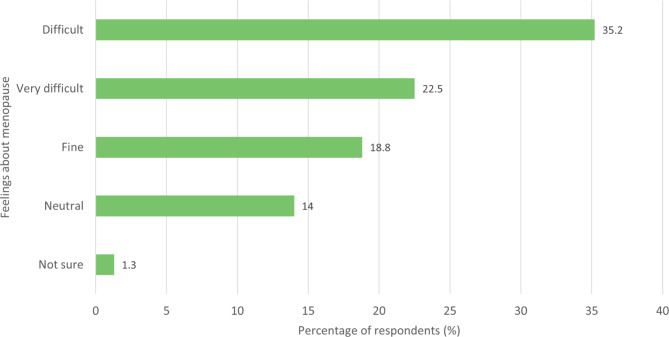
- Feelings of post menopause group towards the menopause

Respondents in the post menopausal group were asked ‘Now that you have been through the menopause, how do you feel about it?’ (Fig. 9) The most common answer was ‘It was difficult’ (35.2%, 329/934), followed by ‘It was very difficult’ (22.5%, 210/934).

**Fig. 10:**
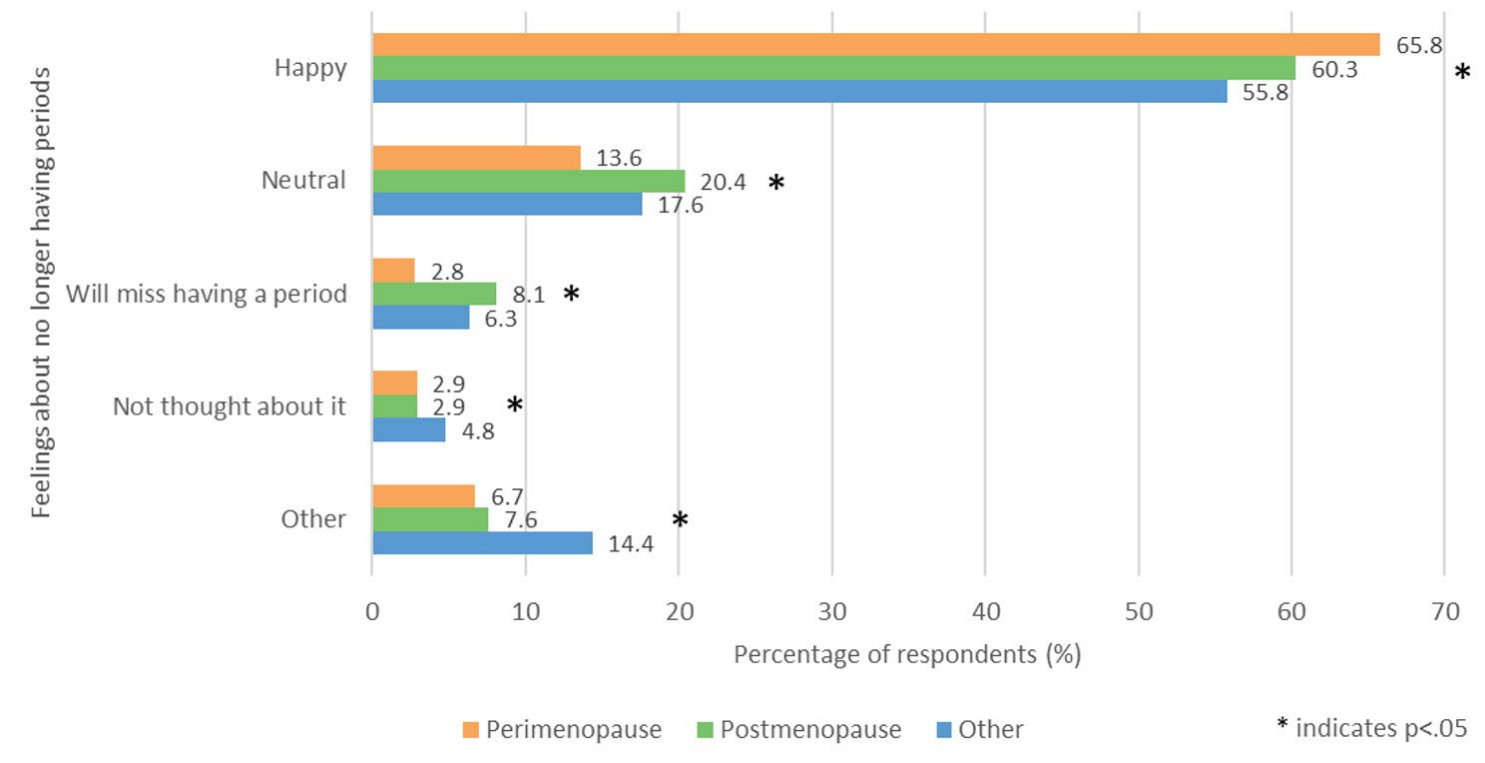
- Feelings about no longer having periods

Respondents were asked ‘What are your thoughts about no longer having periods?’ (Fig. 10) The most common answer in all groups was ‘Happy,’ followed by ‘Neutral.’ Those in the perimenopausal group were significantly less likely to respond ‘Will miss having a period’ (2.8%, 77/950) compared with the other two groups (p < .05). Those in the other group were significantly more likely to respond ‘Not thought about it’ (4.8%, 50/1049) and ‘Other’ (14.4%, 151/1049) compared with the other two groups (p < .05).

Women who selected ‘Other’ after being asked ‘What are your thoughts about no longer having periods?’ (Fig. 10) were then allowed to elaborate on their feelings in their own words (n = 225). These answers revealed conflicting and bittersweet feelings about no longer having periods. Thematic analysis identified four main themes: relief from the burden of periods, sadness of ageing, loss of fertility and loss of identity.

### Relief from the burden of periods

Women generally felt a great sense of relief that they would no longer have to deal with the inconvenience and physical discomfort caused by periods. This was especially true for women with uncommonly heavy and painful periods.

*Have endometriosis. Will be ecstatic when the misery ends.*

*I have suffered from painful and heavy periods my whole life. Part of me looks forward to not having to deal with them anymore.*

*I won’t miss the pain and inconvenience…*

### Sadness of ageing

Many women saw no longer having periods as a marker of ageing, and felt sadness at the prospect of losing their youth.

*…It means I am old, washed up, invisible. It’s closing the door on any semblance of youth and I don’t want that.*

*…Psychologically as a sign of aging it feels like the beginning of the end.*

### Loss of fertility

A large number of women expressed deep sadness and grief at the prospect of losing their fertility. This feeling of loss was particularly profound in childless women, who felt that no longer having periods represented the end of their chances to conceive.

*I’m sad that I never had children and soon the menopause will signal that I will now never have the chance.*

*A bit difficult, as it symbolises the definite end of fertility. As someone who wasn’t able to have children, there is a certain amount of sadness that comes with this.*

### Loss of identity

Some women associated no longer having periods with a loss of identity.

*Feel like loosing [*sic*] my identity, femininity, my connection to my body. Utterly lost, grieving, confused, totally helpless. Struggling every day with mental health.*

*I feel it is the end of who I have been. It will be hard to identify as someone without periods…*

*It’s daunting, feels like becoming a non person.*

## Discussion

The aim of this project was to explore and compare women’s knowledge and attitudes towards the menopause to identify ways in which to improve menopause education and ease the menopause transition.

### Menopause knowledge and education

The survey found that women’s knowledge of the menopause was limited. The majority of women did not feel informed at all or only had some knowledge of the menopause before the age of 40. This is congruent with the growing body of evidence indicating that women generally have a low level of knowledge pertaining to the menopause and its treatments [13–17]. This is concerning as although menopausal symptoms can worsen quality of life (QoL), a better knowledge of the menopause is associated with a greater QoL [18]. Interestingly the post-menopausal women felt they were significantly more informed than the other groups, probably because they were through the perimenopause and had learnt along their way.

When asked when they had first started thinking about the menopause, the most common age range for answers was between 40 and 49. This suggests that many women only begin to think about the menopause when they near the age of experiencing it. Reinforcing this, many women stated that they had only first started thinking about the menopause when symptoms began, or in cases of induced, or early menopause. This indicates a lack of preparedness for the menopause which is likely to make the process more overwhelming and psychologically taxing. Since many women do not know what symptoms are caused by the menopause, this lack of knowledge will certainly lead to delays in diagnosing the perimenopause and delays in advice and treatments.

The majority of respondents felt that the menopause should be taught at school. Despite this, the overwhelming majority of women in all three groups had failed to receive any menopause education at school, with postmenopausal women (who tended to be older than the other two groups) being significantly less likely than the other two groups to have received even basic menopause education. This highlights the failure of the education system to provide women with necessary and desired information about their own bodies. It is therefore promising that the topic of menopause was introduced to the UK school curriculum in 2019, although the quality and long-term impact of this education remains to be seen [19]. Teaching the menopause in school is particularly advantageous as it introduces the topic to both pupils from an early age. Hopefully it will help them understand when their mother and teachers go through the perimenopause. Additionally, it has the potential to reach almost every member of the population, as full-time education is compulsory in the UK [20]. However, it should be noted that many countries worldwide have low rates of school attendance, especially among girls, thus limiting the effectiveness of school menopause education in these areas [21].

The second most common answer from all three groups was that the menopause should be taught in a doctor’s surgery. This may reflect a high level of trust in the medical profession and is consistent with the major role of physicians to provide patient education. However, there are issues involved with this method of menopause education. As the average General Practitioner (GP) appointment in the UK is limited to only 9.2 min, it is unlikely that patients will be able to gain a comprehensive understanding of the menopause in this time [22]. One survey of healthcare professionals found that only half had received any menopause training and only 66% felt confident in managing the menopause [23]. Our perimenopause paper found that many women felt distressed, confused and angry at their GPs’ lack of knowledge of the menopause and its treatments [4]. Until sufficient menopause education is provided to clinicians and other healthcare workers, it is impossible for them to pass on the necessary information to their patients. Menopause education in a doctor’s surgery is also likely to occur much later in life, as most women will only make an appointment to discuss the menopause when they begin to experience symptoms. Additional ways to deliver menopause education need to be considered, such as a women’s wellness clinic and during routine check ups such as at the smear test or mammogram clinic.

The perimenopausal group felt that the menopause should be taught via apps, such as period trackers and fertility apps. It is likely that this disparity is associated with the younger age of the perimenopause group, as the usage of both smartphones and fertility tracking apps (FTAs) has been shown to decrease with age [24, 25]. In line with this finding, the survey found that the perimenopause group was significantly more likely than the postmenopausal group to have sought menopause information via digital sources, such as websites, social media and YouTube. This suggests a need for a shift in the way that menopause information is delivered. With the huge increase in the use of telehealth as a result of the COVID-19 pandemic, this highlights the benefits of delivering healthcare information online [26]. Although menopause apps exist, there is currently little to no research evaluating their impact.

Among the benefits of online menopause education is the ease of accessibility of this information from home. This is particularly beneficial for people who struggle to leave the house, such as full-time carers and those with mental or physical disabilities. The immediate availability of online information also alleviates the delays associated with making and waiting for an in-person consultation, with a 2021 report finding that 16% of patients waited a week or more for a GP appointment [27]. It may therefore reduce the number of unnecessary appointments made, thus reducing pressure on overstretched National Health Service (NHS) services. Online resources can quickly and easily be reviewed at any time; this is extremely valuable for education as studies have shown that 40–80% of medical information provided by healthcare practitioners is immediately forgotten, and almost half of what is remembered is incorrect [28]. Visual aids and translation software can make online information easier to understand for a range of recipients. Finally, online information can be accessed by anyone; in the context of menopause education, this allows younger women and men to be exposed to information that they would not otherwise seek out.

Despite its many merits, there are limitations of providing menopause education online. This mode of delivery is far less personalised than a face-to-face appointment or lecture and does not account for individual circumstances. The lack of direct communication between the creator of the resource and the user creates a risk that information may be misinterpreted, potentially having a detrimental impact on their understanding of the menopause. Although technology has become a ubiquitous part of society, people from lower-income communities, both in the UK and worldwide may not have access to online information. Furthermore, older people or those with learning disabilities may lack the technological proficiency required to utilise online resources.

A major issue is that some of the information online is not accurate and may have no scientific foundation. Women do report using professional society web sites and this should be encouraged, such as the British Menopause Society and their patient arm, Women’s Health Concern. The Royal College of Obstetrics and Gynaecology also has a section ‘for the public’. But both perimenopausal and postmenopausal women in the survey were more likely to have sought menopause information from other websites than official websites. This creates concerns about the quality and reliability of information received due to the high prevalence of health misinformation online, with only approximately 50% of medically-related sites having their content reviewed by doctors [29, 30]. Exacerbating these concerns is the fact that false information online has been shown to spread faster and further than the truth [31]. The reasons for women’s preference for unofficial websites is unclear; however, it is possible that this stems from a lack of awareness of official sources. Additionally, the use of medical jargon may discourage non-healthcare professionals from using official websites. To alleviate this, official websites providing menopause education to the public should ensure that information is delivered in a clear, concise and comprehensible way by engaging the public in their design.

### Management of menopausal symptoms

Over half of the peri and post menopausal women had spoken to a health professional (51.2% and 57.7% respectively). Similarly, approximately half of the women surveyed in a recent Italian study had sought help for the menopause from the Italian National Health System [32]. Conversely, the majority of those in the other group had not done so.

Research suggests that public trust in the medical profession has declined over recent years and it is also well-documented that female patients often receive inferior healthcare due to gender bias exhibited by clinicians [33, 34]. Hence, women may be reluctant to see a healthcare professional due to general mistrust or fear of discrimination. Echoing this, our perimenopause paper found that a number of women were sceptical of their GP or the NHS in general in regards to menopause guidance [4]. Additionally, the menopause is still a taboo topic for many people and can be associated with social stigma in certain communities [35]. Many patients, especially older adults, shy away from discussing sexual health issues with physicians [36, 37]. Therefore, it is possible that a significant proportion of perimenopausal or menopausal women could benefit from the help of a healthcare professional, but choose not to seek one out.

The peri and post menopausal group had mainly used exercise to alleviate menopause symptoms. This is encouraging, as exercise is not only associated with a greater QoL in menopausal women and decreased menopause symptoms, but also general health benefits such as reduced cancer risk, improved cardiovascular health and improved mental health [38–43]. A number of forms of exercise are free and do not require specialist equipment or training (for example walking or running), making it a highly accessible method of menopause management. In our survey asking women if cold water swimming affects menopause symptoms, we found that many reported positively (in preparation). Additionally, the internet provides an abundance of information on exercise, with the NHS website providing exercise guidelines for different age groups and YouTube hosting over 30 million fitness videos [44, 45].

Perimenopausal women were significantly more likely than postmenopausal women to manage symptoms with exercise or nutrition changes such as reduced alcohol or caffeine intake, and significantly less likely to use HRT. This suggests that younger, perimenopausal women may have a greater preference for lifestyle changes and other non-hormonal methods to treat menopausal symptoms. A potential explanation for this observation would be the widespread controversy over the use of HRT which began in the 2000s, largely in part due to the publication of the preliminary results of a Women’s Health Initiative (WHI) study examining the effects of combined oestrogen and progestin hormone therapy [46] which has now been overturned. The current NICE guidelines recommend the use of HRT to alleviate menopausal symptoms [47]. Despite this, public opinion on HRT is mixed and its use remains low, suggesting that many women still have doubts regarding its safety [48].

Additionally, research shows that a large number of women have a poor knowledge of HRT, with a 2020 study by Baquedano et al. [13] finding that a lack of information was the main reason for rejecting menopause hormone therapy [16, 17, 49]. Our perimenopause paper also found that many women struggled to get a prescription for HRT for up to ten years due to their GPs’ lack of knowledge [4]. Many women who are able to get an HRT prescription fail to adhere to the medication due to lack of motivation, or side effects such as headaches, breast tenderness and gastrointestinal disturbances [50, 51].

### Attitudes towards the menopause

There were notable differences between women’s attitudes towards the menopause based on their menopause status. The other group generally had the most negative attitude towards the menopause, with the most common response in this group being that they were dreading it. The perimenopause group and postmenopausal group had similar attitudes overall; compared with the postmenopausal group, the perimenopause group was significantly more likely to be accepting or dreading, but significantly less likely to be neutral. Our menopause survey in the under 40s also found a negative narrative towards the menopause [5]. These findings are consistent with other works which show that postmenopausal women generally have more positive attitudes towards the menopause than premenopausal women [52–54].

There are a number of factors which may contribute to premenopausal women’s negative attitudes towards the menopause. One of the major factors is the media’s portrayal of menopause. Several UK documentaries have only reported negative stories. In print media, negative stories, including those by high profile people and celebrities, are more often reported than positive stories, leading to a lack of balance. It has been said that those reporting positive menopause stories are medically gaslighting and undermining those who have a negative experience.

It is possible that a lack of knowledge contributes to a fear of the unknown as women approaching the menopause are unsure what to expect until they begin to experience it for themselves. This aligns with studies which show that greater knowledge of the menopause is associated with more positive attitudes [16, 55]. Compounding this issue is the societal stigma and shame associated with the menopause. Not only does this promote negative attitudes, but can also exacerbate the issue of insufficient education by posing a barrier to women gaining knowledge of the menopause [35].

Over half of postmenopausal women said that they had found the menopause difficult, or very difficult. This aligns with numerous studies which show that the menopause can decrease quality of life, sexual wellbeing and cause issues in the workplace [18, 56, 57]. Unsurprisingly, there is evidence to suggest that worse menopausal symptoms contribute to more negative attitudes towards the menopause [52, 58].

The survey found that one aspect of the menopause which women generally had positive attitudes was the onset of amenorrhoea. The majority of women in all three groups described themselves as feeling happy at the thought of no longer having periods; this finding has also been observed in similar studies [52, 59]. However, one of the four themes identified in the qualitative analysis was loss of fertility, which women strongly associated with periods ceasing. Yet, what most women failed to acknowledge was that fertility decline begins many years before the menopause. This was perhaps suggestive of a lack of understanding that female fertility declines rapidly from the mid-30s [60]. This would be consistent with numerous studies which highlight the lack of awareness of age-related fertility decline in women [61, 62]. By placing emphasis on menstruation as the main marker of fertility, rather than age, women may delay childbearing until too late in life, hence compromising their ability to successfully conceive [63]. Additionally, advanced maternal age is a risk factor for pregnancy, being associated with chromosomal abnormalities, preterm birth, ectopic pregnancy, foetal loss and maternal mortality [64–67]. Exacerbating this, high-risk pregnancies themselves are likely to provoke fear and anxiety during the gestation period, especially in the era of the COVID-19 pandemic [65, 68].

It is therefore important that women are made aware of fertility decline with age so that they may take appropriate family planning measures and maximise the likelihood of achieving their intended reproductive outcomes. The International Fertility Education Initiative was founded to improve fertility awareness through education, for example by displaying fertility awareness posters in doctor’s surgeries, family planning clinics and schools [4]. Encouraging women to have children earlier in life may reduce the number of involuntarily childless perimenopausal and menopausal women, thereby mitigating some of the negative emotions induced by the onset of amenorrhoea. Nonetheless, it is likely that this aspect of the menopausal transition will always elicit certain negative emotions due to the association of periods with youth and identity, as identified in the qualitative analysis and in other studies [59].

Not only do worse menopausal symptoms contribute to negative attitudes of women towards the menopause, but it has been suggested that negative attitudes themselves can exacerbate menopausal symptoms, thus having significant implications on health [11, 52, 58, 69]. Additionally, there is evidence to suggest that husbands’ attitudes towards the menopause can also influence the severity of women’s symptoms [69, 70]. This highlights the importance of providing menopause education and promoting positive attitudes to both men and women alike.

### Limitations

The majority of respondents in the survey were white, heterosexual, university postgraduates living in the UK, thus compromising diversity. To overcome this, we currently have the same survey live, specifically aimed at Black women and are working with people who work in this area to promote this survey. We aim to repeat this for Asian women. The age of respondents was limited to those 40 or over and we have undertaken a survey of women under 40 to hear their views [3]. Men were not included in the survey, despite the fact that their attitude and knowledge towards the menopause can have a major impact on women’s experiences of the menopause. Additionally, transgender men and gender diverse people may also undergo menopause but were not included in this survey as they would require a different set of questions. The promotion of the survey on social media created a risk of selection bias towards followers of the people promoting the survey. There was also a risk of recall bias for certain questions; for example, when asking women the age at which they had started to think about the menopause, as for some, this may have required them to think back many years into the past. The analysis was dependent on respondents’ self-identification as perimenopausal, postmenopausal or other; although menopause can generally be self-diagnosed, there was a risk that some women were incorrect about their own menopause status, hence skewing the results. The other group of women would have possibly consisted of a mix of all the following - peri menopausal women who did not know they were in peri menopause, postmenopausal women who did not correctly understand they were post menopausal, women who were still continuing with regular menstrual cycles and those of different forms of hormonal contraception which could have altered their symptoms and menstrual pattern.

## Conclusion

It is important that we ensure women and health professionals understand the perimenopause transition, its symptoms and treatments. But it is also key that we create a more positive view to the menopause, especially life post menopause, which many women find as the most rewarding time of their lives. Both of these issues can be solved with effective menopause education.

### Electronic supplementary material

Below is the link to the electronic supplementary material.

**Additional File 1:** The Survey

## Data Availability

Due to UCL ethics approval, the data is not available beyond UCL. For enquiries, please contact joyce.harper@ucl.ac.uk.
